# A Snapshot of Microbial Succession and Volatile Component Dynamics of Marselan Wine in Xinjiang During Spontaneous Fermentation

**DOI:** 10.3390/foods14060994

**Published:** 2025-03-14

**Authors:** Qingquan Fu, Fangfang Wang, Tiantian Tang, Zimen Liu, Lilin Wang, Qingling Wang, Xuewei Shi, Bin Wang

**Affiliations:** 1 Food College, Shihezi University, Shihezi 832000, China; fu1599105353@163.com (Q.F.); wff200905@163.com (F.W.); 15124534465@163.com (T.T.); liuzimenglemon@163.com (Z.L.); qingling1100@163.com (Q.W.); shixuewei@shzu.edu.cn (X.S.); 2 Key Laboratory of Characteristics Agricultural Product Processing and Quality Control (Co-Construction by Ministry and Province), Ministry of Agriculture and Rural Affairs, School of Food Science and Technology, Shihezi University, Shihezi 832000, China; 3 Key Laboratory for Food Nutrition and Safety Control of Xinjiang Production and Construction Corps, School of Food Science and Technology, Shihezi University, Shihezi 832000, China; 4 Engineering Research Center of Storage and Processing of Xinjiang Characteristic Fruits and Vegetables, Ministry of Education, School of Food Science and Technology, Shihezi University, Shihezi 832000, China; 5 Production & Construction Group Key Laboratory of Special Agricultural Products Further Processing in Southern Xinjiang, College of Food Science and Engineering, Tarim University, Alar 843300, China; 120060036@taru.edu.cn

**Keywords:** wine, spontaneous fermentation, microbial communities, volatile composition, correlation analysis

## Abstract

Marselan wine is characterized by distinctive flavors of blackcurrant, cranberry, and spice, which are significantly influenced by environmental factors such as region and climate. In this study, we analyzed the dynamic changes in the microbial community, physicochemical indices, and flavor compounds during the spontaneous fermentation of Marselan wine in Xinjiang using high-throughput sequencing (HTS), high-performance liquid chromatography (HPLC), and headspace solid-phase microextraction gas chromatography–mass spectrometry (HS-SPME-GC-MS). The results indicated that the sugar content decreased from 259.12 g/L to 22.45 g/L, while the ethanol content increased to 13.63 ± 0.15% vol after 12 days of fermentation. The predominant aromatic components identified in Marselan grapes include isophorone, 2,3-pentanedione, 2-hexenal, and melonal. After fermentation, ethanol, phenethyl alcohol, isoamyl acetate, ethyl acetate, and ethyl hexanoate were produced, imparting rose, cream, and fruit flavors to wine. The key microorganisms involved in the spontaneous fermentation of Marselan wine include *Saccharomyces*, *Starmerella*, *Pichia*, *Pseudomonas*, *Sphingomonas*, and *Aspergillus*. These microorganisms contributed substantially to the main physicochemical indices and flavor profiles. *Saccharomyces* and *Pichia* enhanced the formation of most alcohols and esters, whereas *Aspergillus*, *Acremonium*, and *Fusarium* inhibited the synthesis of numerous volatile compounds. These findings provide valuable theoretical references for improving the quality of Marselan wines in Xinjiang.

## 1. Introduction

Wine is a globally consumed alcoholic beverage derived from complete or partial alcoholic fermentation of fresh grapes or their juices [1]. According to its color, wine can be classified as red, white, and rose wine [2]. Red wine dominates the global wine market because of its appealing color, strong antioxidant capacity, rich aroma, and full-bodied characteristics [3]. Common grape varieties used in dry red wine production include Cabernet Sauvignon, Merlot, Marselan, etc. [4]. Cabernet Sauvignon grapes have been widely used in wine production because of their characteristics, such as ease of cultivation, high tannin content, deep color, complex flavor, and rich style [5]. Furthermore, there is a mature technology in the wine industry, including fermentation conditions, equipment, commercial yeast, exogenous pectinase, and sulfur dioxide [6]. However, reliance on Cabernet Sauvignon and standardized production techniques has led to significant concerns over wine-style homogenization [7]. Consequently, the screening of various distinctive grape cultivars to develop characteristic wines has become a market demand and research hotspot [8]. As one of the distinguishing grape cultivars, Marselan has emerged as the most developed red wine variety in various production regions in China by virtue of its strong disease resistance and high-quality small berries [9]. Marselan wine production and consumption has grown rapidly, elevating it to the status of a rising star in the modern wine market [4].

Wine has retained global popularity for millennia, primarily because of its superior attributes, including pleasant taste, delightful flavor, high nutritional value, and aesthetic color [10]. The taste of wine largely stems from its sugar, acid, alcohol, and tannin contents, which interact intricately [11]. The levels of sugar and acid present in grapes play a crucial role in determining not only the ethanol content of the resulting wine but also its inherent flavor profile [12]. In particular, the composition and content of organic acids are closely linked to the style and quality of wine, impacting on sensory perception, coloration, flavor, and its microbiological and physicochemical stability [13]. In addition to taste, a delightful flavor significantly contributes to attracting consumers toward wine [14]. Flavors in wine include alcohols, aldehydes, acids, esters, and other compounds produced by grape metabolism, microbial activity, and the aging process [15]. These flavors vary with grape quality, cultivar, starter culture, equipment, and processing method [16]. Additionally, wine, especially red wine, possesses significant levels of sugars, organic acids, polyphenols, vitamins, and trace elements, which collectively confer high nutritive value, such as anti-aging and antioxidant effects, and contribute to mitigating cardiovascular diseases [17].

The transformation of grape juice into wine is a multifaceted process involving biochemical reactions that occur in the presence of complex microbial systems [18,19]. Wine fermentation primarily comprises two processes, alcoholic fermentation and malolactic fermentation, which are completed under the catalytic action of yeast and lactic acid bacteria, respectively [20]. During alcoholic fermentation, *Saccharomyces cerevisiae* converts sugar into ethanol, which is an essential component of wine [21]. At the same time, non-*Saccharomyces cerevisiae*, such as *picha*, *Hanseniaspora*, *Candida*, and *Metschnikowia*, contribute to the formation of various secondary metabolites, some of which are primary flavor substrates in wine [22,23]. In the malolactic fermentation, lactic acid bacteria convert malic acid into lactic acid in wine, which reduces acid, improves stability, and produces numerous flavor substrates [24]. Wine fermentation can be categorized as either inoculation or spontaneous fermentation based on the origin of the starter culture [25]. In inoculation fermentation, a specific quantity of commercial yeast is added to grape juice to initiate fermentation [15]. This method is characterized by a rapid fermentation speed, ease of process control, and a stable fermentation process, although this often results in relatively uniform flavor profiles [26]. In contrast, spontaneous fermentation relies on native microorganisms present on grape skins [27]. Although this approach is characterized by slower fermentation rates, challenges in process control, and greater variability, it frequently leads to a more complex and rich flavor profile [28]. The isolation of various yeasts involved in the formation of wine flavors by investigating the dynamic changes in microorganisms and flavors in wine has become a research focus in the development of wines with complex flavors and regional characteristics [23].

Marselan grapes, which exhibit strong adaptability and superior fermentative characteristics in Xinjiang, possess considerable development potential [29]. In recent years, the cultivation of Marselan grapes has expanded rapidly, owing to the excellent fermentative characteristics of Marselan wine [30]. Compared to general wine fermentation, productive practices indicate that spontaneously fermented Marselan wine exhibits superior quality; however, the underlying fermentation mechanisms remain unclear. In this study, dynamic changes in the microbial community, physicochemical indices, and flavor compounds in Marselan wine during spontaneous fermentation in Xinjiang were analyzed using high-throughput sequencing (HTS), high-performance liquid chromatography (HPLC), and headspace solid-phase microextraction gas chromatography–mass spectrometry (HS-SPME-GC-MS) technology. Moreover, we identified the core flavor compounds in wine along with their associated microorganisms using multivariate data analysis. These findings enhance our understanding of the spontaneous fermentation mechanisms in Marselan wine and provide a theoretical foundation for standardizing wine production and improving wine quality.

## 2. Materials and Methods

### 2.1. Spontaneous Fermentation and Sample Collection

In 2023, Marselan grapes from the professional winery Great Wall Wine in Xinjiang, China (86.13°E, 44°18′ N), were obtained for the present study. Fresh and ripe Marselan grapes were harvested using scissors that had been sterilized with 75% alcohol. All the grapes were stored at 4 °C and transported to the laboratory within 8 h. A portion of the sample was cryopreserved at −80 °C as a contingency. The biochemical composition of these grapes revealed a total sugar content of 258 g/L, total acid of 5.5 g/L, pH of 3.98, and soluble solid content of 26.5 °Bx.

Before fermentation, the fresh grapes were manually pruned, crushed, and transferred into a clean, decontaminated 10 L fermentation tank. Pectinase (30 mg/L) was added, followed by potassium metabisulfite, to the fermentation broth to achieve a total sulfur dioxide (SO2) concentration of around 40 mg/L, thereby preventing interference from non-beneficial bacteria during the fermentation process. Spontaneous fermentation was conducted at 22 ± 0.5 °C in triplicate, without incorporating any commercial strain. On days 0, 3, 6, 9, and 12 of the fermentation, 100 mL wine samples were gathered and labeled as A, B, C, D, and E, respectively. Each sample underwent centrifugation at 8000× *g* for 10 min at 4 °C. The sediment was collected for high-throughput sequencing analysis, and the supernatant was utilized to assess the physicochemical properties and volatile compounds [31].

### 2.2. Physicochemical Properties Determined During the Fermentation Process

Throughout the spontaneous fermentation process of Marselan wine, the total sugar, total acid, ethanol content, and pH were systematically measured. The total sugar content was determined using the 3,5-dinitrosalicylic acid method. The total acid and ethanol contents were quantified according to the national standard GB/T 15038-2006, titled “General Analytical Procedure for Wine and Fruit Wine” [32]. The pH was recorded using a calibrated pH meter.

The contents of organic acids and soluble sugars were quantified by high-performance liquid chromatography (HPLC), following previously reported methods with minor modifications [25,33]. For organic acid measurement, the wine samples underwent centrifugation and were subsequently filtered into injection vials by 0.45 µm filters. Chromatographic analysis was conducted on a Dikma C18 column (5 µm, 4.6 mm, 250 mm; Diamonsil Plus Technology, Hangzhou, China) maintained at 40 °C. The mobile phase consisted of 0.1% phosphoric acid in water (0.015 mol/L) and methanol (99.99%) at a flow rate of 1 mL/min, and detection was performed at 210 nm using a UV detector. A standard curve was constructed using standard samples to establish the correlation between peak area and concentration. To determine soluble sugars in wine, chromatographic separation was carried out utilizing an XBridge amide column (5 µm, 4.6 mm × 250 mm) with acetonitrile (99.99%) and water as the mobile phases at a flow rate of 1 mL/min. An injection volume of 20 µL was used, the column’s temperature was maintained at 30 °C, and elution peaks were monitored with an RID-10A differential detector. Each measurement was performed in triplicate.

### 2.3. Volatile Compound Analysis

The contents of volatile compounds were detected using previously described methods with appropriate modifications [34]. In brief, 5 mL of wine sample was transferred into a 25 mL headspace injection vial containing 1 g of sodium chloride and 2 μL of the internal standard 3-octanol (330 µg/L). A solid-phase microextraction fiber (DVB/CAR/PDMS 50/30 µm; Supelco, Bellefonte, PA, USA) was then inserted into the headspace injection vial and extracted in the headspace at 40 °C for 40 min. Following the extraction process, the fibers were removed and inserted into the inlet of a gas chromatography (GC) column (HP INNOWAX column, 30 m × 0.25 mm; Agilent, Shanghai, China), where they underwent desorption at 210 °C for 5 min. The inlet temperature was set to 230 °C while utilizing helium as the carrier gas at a flow rate of 1 mL/min and an electron energy of 70 eV. The temperature program proceeded as follows: an initial phase held at 40 °C for 5 min, then heating to 100 °C at a rate of 3 °C/min, followed by a 25 min run. Subsequently, the temperature rose to 180 °C at a rate of 5 °C/min, followed by a 41 min hold at 180 °C, and finally it was heated to 230 °C at a rate of 10 °C/min, maintaining this temperature for an additional 5 min. Compound identification was achieved through computer-assisted matching with the NIST 14 database, and a semi-quantitative method was utilized to ascertain the content of volatile compounds.

### 2.4. Analysis of Microbial Diversity

The wine samples were centrifuged at 8000 rpm for 10 min at 4 °C, and the precipitates were collected and processed into a fine powder in a mortar using liquid nitrogen. Total genomic DNA was extracted using the E.Z.N.A. Soil DNA Kit (Omega Biotek, Norcross, GA, USA). Spectrophotometry and agarose gel electrophoresis were employed to evaluate the quantity and quality of the extracted DNA samples, respectively. These DNA samples served as templates to amplify the V3–V4 hypervariable regions of the bacterial 16S rRNA gene using the primers 338F (5′-ACTCCTACGGGAGGCAGCAG-3′) and 806R (5′-GGACTACHVGGGTWTCTAAT-3′). For fungal analysis, the internal transcribed spacer (ITS) region (ITS5-ITS2) was amplified using the primers ITS5 (5′-GGAAGTAAAAGTCGTAACAAGG-3′) and ITS2 (5′-GCTGCGTTCTTCATCGATGC-3′). PCR amplification was conducted as follows: pre-denaturation at 95 °C for 5 min; followed by 36 cycles of denaturation at 95 °C for 45 s, annealing at 55 °C for 30 s, and extension at 72 °C for 50 s; and a final extension at 72 °C for an additional 10 min [35]. The products amplified by PCR were sequenced utilizing an Illumina MiSeq platform (Shanghai Personal Biotechnology Co. Ltd, shanghai, China). Paired-end sequencing (2 × 250 bp) was conducted on an Illumina MiSeq 2500 platform, in accordance with standard protocols. The quality and concentration of the constructed library were assessed using Agilent 2100 (Agilent Technologies, Santa Clara, CA, USA) and QuantiFluor (Promega Corporation, Madison, WI, USA). Raw reads underwent de-priming, quality filtering, denoising, splicing, and de-chimerization using QIIME2 (2019.4). Amplicon sequence variants (ASVs) were clustered at 100% similarity using DADA2 in the QIIME2 2019.4 software. Subsequent microbial bioinformatic analyses were performed based on the abundance distribution data from various wine samples.

### 2.5. Statistical Analysis

All wine samples underwent triplicate analysis, and the results are presented as the mean ± standard deviation. One-way analysis of variance (ANOVA) followed by Duncan’s multiple range test (*p* < 0.05) was performed using SPSS 22.0 to determine significant differences among samples. Histograms and chord diagrams representing volatile compounds were generated using Origin 2021 software. TBtools 2.136 was used to draw heat maps and Venn diagrams. Additionally, two-way orthogonal partial least squares (O2PLS) analysis was performed using SIMCA 14.1 to identify and screen the main flavor compounds and microbiota. In addition, multifactorial analysis was conducted with SIMCA 14.1 software to evaluate the interactions between microbial communities and volatile compounds; the resulting data were visualized through Cytoscape (version 3.10.2).

## 3. Results and Discussion

### 3.1. Dynamics of Physicochemical Properties and Flavor Substances During Fermentation

#### 3.1.1. Analysis of Physicochemical Characteristics

**Figure 1:**
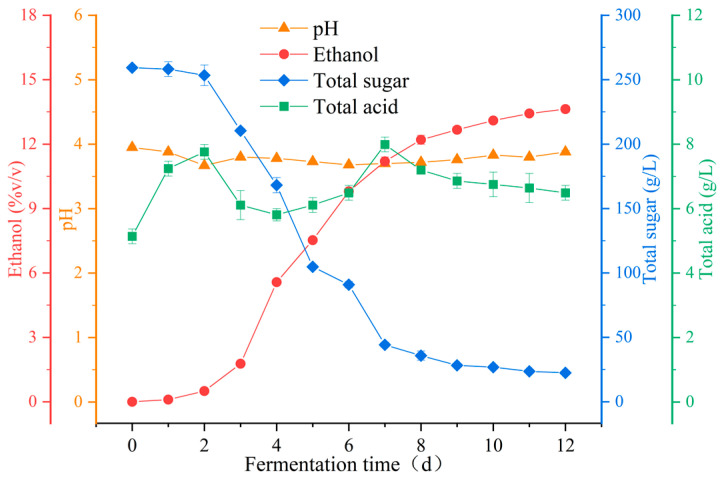
Dynamics of physical and chemical indicators during spontaneous fermentation of Marselan wine.

Physicochemical indices are fundamental indicators for evaluating wine quality, and their formation primarily depends on the grape cultivars, growing conditions, and vinification process [36]. Dynamic variations in the pH, total sugar, total acid, and ethanol contents were monitored for 12 days during the spontaneous fermentation of Marselan wine (Figure 1). Throughout the fermentation process, the pH values fluctuated within the range of 3.67–3.95, whereas total acid varied from 5.13 to 7.99 g/L and was ultimately maintained around 6.49 g/L at the conclusion of fermentation. Concurrently, the total sugar concentration showed a significant decrease from 259.12 to 22.45 g/L. In the initial phases of wine fermentation, *Saccharomyces* rapidly multiplied through glucose consumption in the fermentation broth, and the total sugar content in wine declined markedly once the yeast population reached a certain threshold. As the fermentation progressed, the *Saccharomyces*-mediated sugar-to-alcohol conversion process led to a significant increase in the ethanol concentration, ultimately reaching 13.63 ± 0.15% (*v*/*v*) on day 12.

#### 3.1.2. Organic Acid Composition

**Figure 2:**
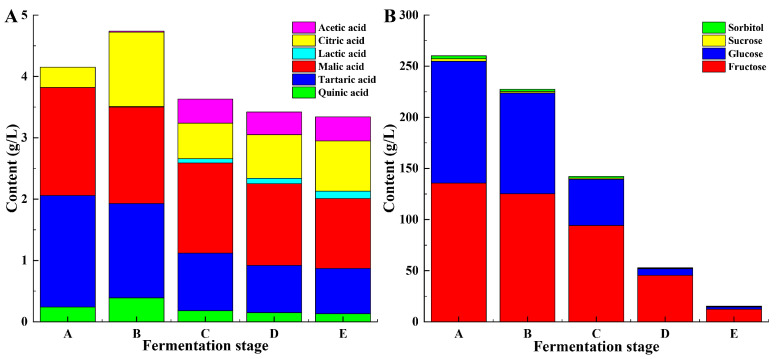
Contents of the organic acids (**A**) and sugar composition (**B**) at different fermentation stages. A, B, C, D, and E represent samples collected on fermentation days 0, 3, 6, 9, and 12, respectively.

Organic acids significantly affect the astringency, flavor, and stability of wine, thereby exerting a critical influence on its overall quality [37]. In this study, HPLC analysis identified six organic acids (lactic acid, acetic acid, citric acid, malic acid, tartaric acid, and quinic acid) produced during spontaneous fermentation (Figure 2A). The proportion of these acids shifted over the course of the fermentation. Consistent with Xu et al. [22], malic acid and tartaric acid were predominant organic acids in grape, accounting for >85% of the total content. As fermentation progressed, both the tartaric and malic acid concentrations decreased, whereas some malic acid was metabolized by lactic acid bacteria to lactic acid [20]. Acetic acid was mainly generated during the mid-fermentation stage, with minor fluctuations thereafter. After fermentation, the composition of organic acids in the wine was as follows: quinic acid (3.89%), tartaric acid (22.16%), malic acid (34.13%), citric acid (24.55%), acetic acid (11.68%), and lactic acid (3.59%). These variations in the organic acid composition confer distinct sensory attributes to wine. Furthermore, factors such as grape cultivars, climate, soil, and fruit maturity can significantly influence organic acid levels and, consequently, the overall quality of the wine [38].

#### 3.1.3. Sugar Composition

Marselan grapes contain notably high levels of sugar, which directly influence yeast proliferation and metabolic activity, thereby shaping the quality of the resultant wine [22]. The composition and concentration of soluble sugars were quantified using HPLC (Figure 2B). Prior to alcohol fermentation, the fructose, glucose, sucrose, and sorbitol concentrations were 135.38, 118.12, 2.42, and 2.09 g/L, respectively. Among all grape samples, glucose and fructose were identified as the predominant sugars present in all the grapes. Glucose and fructose emerged as the most abundant sugars. Hexose serves as the primary energy source for *Saccharomyces cerevisiae*, with glucose being the preferred carbon source [39]. Consequently, glucose was consumed more rapidly than fructose during the initial stages of alcohol fermentation. In addition, trace amounts of sucrose and sorbitol were detected in wine, potentially contributing to its overall stability [40].

#### 3.1.4. Volatile Compounds

The unique flavor of wine is strongly influenced by various volatile compounds. During spontaneous fermentation, HS-SPME-GC-MS analysis identified 59 volatile compounds, including 13 esters, 22 alcohols, 8 acids, 7 aldehydes, 5 ketones, and 4 other aromatic compounds (Figure S1). The total concentrations of these aromatic compounds, particularly those of esters, alcohols, and acids, increased over time (Figure S2). Principal component analysis (PCA) was used to clarify the connections and distinctions among the volatile compounds. Collectively, the 59 identified components accounted for 75.8% of the total variance, where the first principal component (PC1) and the second principal component (PC2) contributed to 51.1% and 24.7% of the total variance, respectively. Notably, the significant difference in volatile profiles between stages A-B and C-E indicated that there was a significant disparity in the volatile components found in grapes compared to those in wine. During spontaneous fermentation, the samples in the PCA score plot shifted from the X-coordinate negative half-axis to the positive half-axis, highlighting the emergence of alcohols and esters as the primary aromatic compounds produced through yeast metabolism and esterification. These compounds effectively characterize the fermentation aroma of wine. During the mid-to-late fermentation stages, the samples in the scoring plot shifted from the Y-coordinate positive half-axis to the negative half-axis, suggesting that the primary flavor profile is largely formed by mid-fermentation, with subsequent modifications occurring from the middle stage to the end of fermentation. These changes showed that the main aroma substances in wine fluctuated throughout the various stages of fermentation. Most volatile compounds were found to accumulate more in the later stages, implying that fermentation may be more conducive to the synthesis of aromatic compounds, particularly alcohols and esters [32].

**Figure 3:**
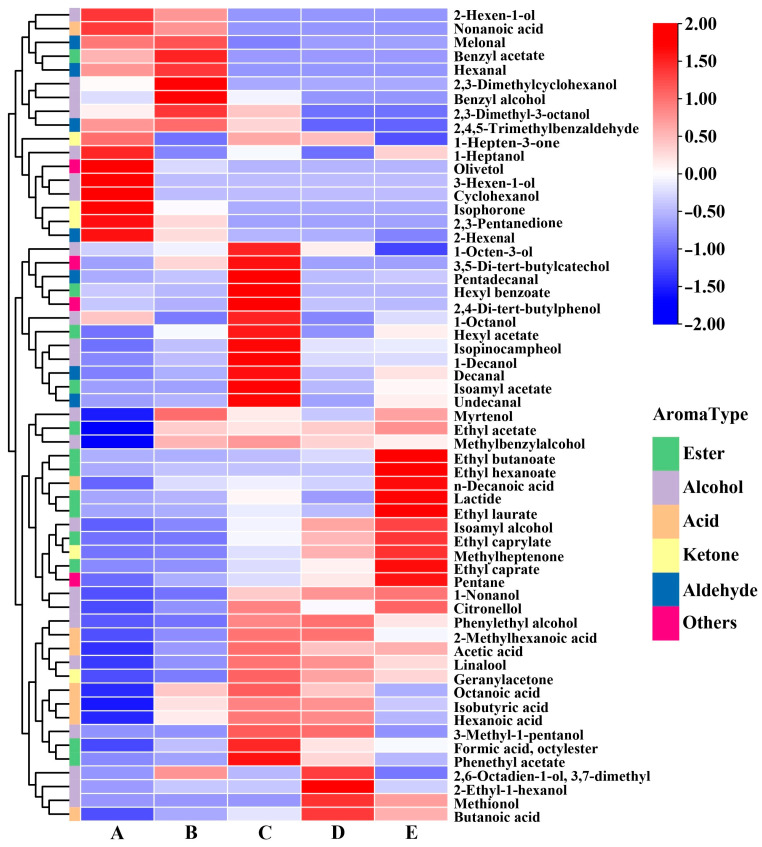
Heatmap cluster analysis of the volatile components identified during fermentation. The colors correspond to normalized mean levels, from low (blue) to high (red). A, B, C, D, and E represent samples collected on fermentation days 0, 3, 6, 9, and 12, respectively.

The dynamic evolution of the volatile compounds is shown in Figure 3 and Table S1. In Marselan grapes, the predominant aroma components were aldehydes and ketones, specifically isophorone, 2,3-pentanedione, 2-hexenal, and melonal, which probably characterize the distinct varietal aroma of Marselan grapes in Xinjiang. Esters are important flavoring substances during wine fermentation [5]. Unlike the study by Lu et al. [30], the primary esters comprised isoamyl acetate, ethyl acetate, phenethyl acetate, and ethyl caproate, which imparted rose, cream, and fruit flavors to the wine [36,41]. The esters that played a key role in the distinctive flavor of wine were predominantly ethyl esters and acetates, which can be synthesized through the esterification of ethanol or acetic acid [42]. Furthermore, microbial cells are capable of producing these compounds through the utilization of acetyl coenzyme A along with higher alcohols as substrates during fermentation facilitated by alcohol acetyltransferases [43]. Alcohols serve as an important source of sweetness while also acting as aroma enhancers in wines [32]. Following spontaneous fermentation, wine exhibits elevated concentrations of phenylethyl alcohol, 3-methyl-1-butanol, 1-hexanol, and benzyl alcohol. Nonetheless, their relatively elevated odor thresholds limit their direct impact on the flavor profile. Acid is an important aromatic substance in wine, while these compounds can produce unpleasant flavors when they exceed the sensory threshold [2]. Consistent with Lan et al. [9], acetic acid was found to be the most abundant volatile acid in wine. The presence of these substances significantly improves the flavor attributes of wine, which probably characterizes the fermentation aroma of Marselan wine.

### 3.2. Microbial Community During Fermentation Process

#### 3.2.1. Microbial Diversity

Microbial diversity and its dynamic changes play a crucial role in determining the quality and properties of wine [19]. HTS was used to investigate the diversity and succession of the microbial communities during fermentation. A total of fifteen wine samples were gathered from five distinct fermentation stages, and Illumina sequencing was used to examine the number of fungal and bacterial taxa across various taxonomic levels. The alpha diversity within the microbial community was assessed through the Chao1 and Shannon indices (Figure S3 and Table S2). As fermentation progressed, bacterial community diversity exhibited a decreasing trend. Interestingly, there was a higher prevalence of unique ASVs in bacteria compared to fungi, indicating that the bacterial diversity exceeded the fungal diversity. For fungi, the Chao1 and Shannon indices decreased during early alcohol fermentation stages and subsequently increased as the fermentation progressed. In contrast, bacterial communities displayed an opposing trend. These changes in diversity indices for both bacteria and fungi could be linked to the microorganisms’ adaptive responses to environmental stressors encountered during alcoholic fermentation [44]. To further characterize the fungal operational taxonomic units (OTUs) at different fermentation stages, 263, 174, 128, 124, and 132 fungal OTUs were detected in A, B, C, D, and E, respectively (Figure S4A). Among these, 21 OTUs were identified as common across all the five fermentation stages. In terms of bacterial OTUs, 233, 233, 276, 232, and 249 were identified in the respective stages, with 14 OTUs common to all the stages (Figure S4B). These findings indicate that the structure of the microbial community in wine varies across the different stages of fermentation.

Overall, the composition of microbial communities in wine must change significantly as fermentation proceeds, resulting in significant variations in the diversity indices for both fungi and bacteria [45,46]. As the fermentation time increased, the diversity of bacterial communities showed a decreasing trend. The phylum Ascomycota was more abundant compared to other phyla, representing 99.07% of the total microbial community. In terms of bacterial composition, the dominant phyla included Proteobacteria, Firmicutes, and Actinobacteriota, which comprised 76.4%, 9%, and 7.16% of the bacteria, respectively (Figure S5).

**Figure 4:**
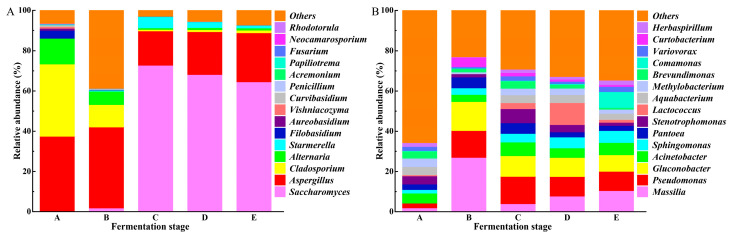
The relative abundances of the top 15 fungi (**A**) and bacteria (**B**) at the genus level. A, B, C, D, and E represent samples collected on fermentation days 0, 3, 6, 9, and 12, respectively.

To investigate shifts in microbial genera during wine fermentation, the top 15 genera based on average relative abundance were selected. At the genus level, the fungal community was predominantly composed of *Saccharomyces* (47.18%), *Aspergillus* (31.92%), *Alternaria* (4.99%), and *Starmerella* (2.36%) (Figure 4A); these results were different from those reported in Lu et al. [30]. During the fermentation process, the rapid proliferation of brewing yeast significantly inhibited the growth of certain fungal microorganisms [47]. Compared to the yeast genera, the alcohol fermentation ability of non-yeast genera was less pronounced. *Aspergillus*, *Cladosporium*, and *Alternaria* were identified as the primary fungal populations in grape skin. Additionally, *Alternaria* has been observed as the dominant fungal genus in Merlot, Pinot Noir, Syrah, and Cabernet Sauvignon grapes in Xinjiang [22].

The diversity of bacteria present during wine fermentation exceeded that of fungi [48]. The major bacterial genera were *Massilia* (10.17%), *Pseudomonas* (9.71%), *Gluconobacter* (8.51%), and *Pantoea* (3.68%) (Figure 4B). The relative abundance of *Gluconobacter*, a common acetic acid bacterium on grape skins, increased as fermentation progressed. *Lactococcus* began to proliferate in the middle of fermentation and peaked in later stages. The low levels of *Gluconobacter*, *Lactococcus*, and other bacteria in the early stages may be attributed to the addition of SO_2_ at this stage [49]. *Pantoea*, an endophytic bacterium frequently found on plant surfaces and fruits, was prevalent at stage A, suggesting that it originated from the grapes and the surrounding environment. From stage B onward, *Massilia*, *Pseudomonas*, and *Gluconobacter* exhibited higher relative abundances, becoming the primary bacterial genera throughout the wine fermentation process. Meanwhile, *Lactococcus* demonstrated a moderate relative abundance that increased during the later stages of alcoholic fermentation. These microorganisms generally thrive under low-oxygen and low-pH conditions typical of post-fermentation since they are both facultative anaerobes and acid-resistant bacteria [50].

**Figure 5:**
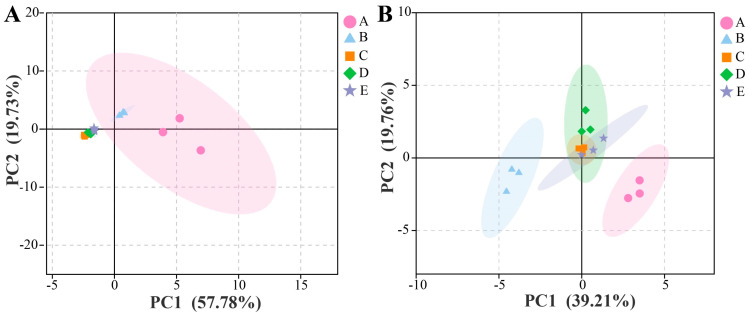
Principal component analysis (PCA) scatter plot of the fungal (**A**) and bacterial (**B**) communities in the samples. A, B, C, D, and E represent samples collected on fermentation days 0, 3, 6, 9, and 12, respectively.

PCA was employed to evaluate the microbial diversity and identify differences and resemblances among microbial communities (Figure 5). The beta diversity analysis of the microbial community structures showed that PC1 accounted for 57.8% of the variance observed in the samples, while PC2 explained 19.7%. In terms of bacterial structure, PC1 and PC2 contributed to 39.1% and 16.8% of the cumulative variance, respectively. The microbial composition of the samples changed significantly as fermentation progressed. Specifically, the microbial community in stage A differed substantially from those in the other stages, whereas the microbiota in stages D and E, which occurred towards the end of fermentation, exhibited similarities. Throughout the fermentation process, the composition of the bacterial communities varied significantly in terms of distribution across different stages, indicating that fermentation time may be the primary factor driving these differences [51].

#### 3.2.2. Co-Occurrence and Exclusion Analyses Among Different Microorganisms

Microbial interactions are significant factors that influence microbial structure and regulate the sensory characteristics of wine [52]. Positive and negative correlation analyses were conducted to elucidate the symbiotic and antagonistic relationships among fungal genera using Pearson’s rank coefficients. The findings indicated that *Saccharomyces* displayed negative correlations with nearly all other fungal genera, with the exception of *Starmerella* (Figure S6A). A comparable pattern was observed for *Starmerella*. Among the prominent fungal genera, *Saccharomyces* demonstrated effective fermentation catabolism and exhibited a strong tolerance to acidic conditions and ethanol, thereby conferring a significant competitive advantage throughout fermentation [53]. Recent ecological research has additionally noted the significant presence of *Starmerella* in high populations alongside *Saccharomyces*, which is consistent with the ability of *Starmerella* to produce relatively high levels of ethanol and thrive in environments with elevated sugar concentrations [54]. In contrast, a positive correlation was observed among all other fungal genera.

Regarding bacterial genera, *Massilia* showed a negative correlation with most other genera, with the notable exceptions of *Pseudomonas*, *Gluconobacter*, *Pantoea*, and *Curtobacterium* (Figure S6B). *Lactococcus* showed weak correlations with the other bacterial genera. Furthermore, a significant exclusion relationship was identified between fungal and bacterial communities (Figure S6C). Specifically, *Saccharomyces* showed exclusion patterns for *Massilia*, *Pantoea*, *Brevundimonas*, and *Curtobacterium*. This suggested that these microbial taxa may experience difficulty adapting to the selective pressures arising from the rising ethanol concentrations and the increased acidity generated by *Saccharomyces* and *Starmerella* [55]. However, *Pseudomonas* and *Gluconobacter* displayed co-occurrence patterns with *Saccharomyces* and *Starmerella*.

### 3.3. Microbial Community Succession Driven by Physicochemical Factors

**Figure 6:**
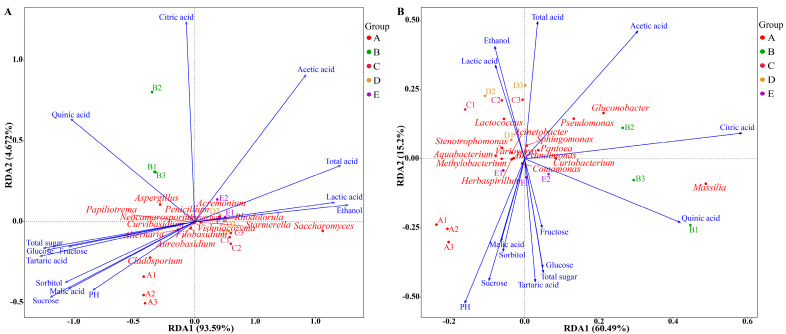
RDA of microorganisms and physicochemical factors in the fermentation of Marselan wine. Correlations between physicochemical factors and fungi (**A**). Correlations between physicochemical factors and bacteria (**B**).

Environmental shifts in the fermentation environment can drive the succession of microbial communities and shape the ecological dynamics within wine communities [56]. Consequently, a Mantel test utilizing Spearman’s correlation coefficient was employed to clarify the possible connections between abiotic factors and the succession of microbial communities. Redundancy analysis (RDA) was employed to assess the influence of various quality indicators (e.g., organic acids, soluble sugars, pH, ethanol content, total sugar, and total acid) on the distribution of fungal and bacterial communities during spontaneous wine fermentation. As shown in Figure 6A, the contribution of RDA1 and RDA2 accounted for 93.59% and 4.67% of the overall variation, respectively, and together accounted for 98.26% of the variation. These results suggested that the analysis could effectively elucidate the connection between the fungal community and quality indices to a considerable extent. Importantly, there were positive correlations observed between *Saccharomyces* and *Starmerella* with total acid, ethanol, and lactic acid, whereas *Alternaria* and *Cladosporium* showed positive correlations with soluble sugars, quinic acid, malic acid, and tartaric acid. Furthermore, a close association was identified between *Aspergillus* and the production of organic acids including citric acid and quinic acid [57].

Regarding the bacterial community (Figure 6B), RDA1 and RDA2 accounted for 60.49% and 15.2% of the total variation, respectively. *Massilia* and *Curtobacterium* exhibited a positive association with citric acid and quinic acid while demonstrating a negative correlation with ethanol, lactic acid, and acetic acid. In contrast, ethanol, lactic acid, total acid, and acetic acid, which are key contributors to the composition of the predominant species during spontaneous fermentation, showed strong positive correlations with *Lactococcus*, *Stenotrophomonas*, and *Acinetobacter*. Conversely, *Gluconobacter*, *Lactococcus*, *Pseudomonas*, and *Pantoea* were negatively linked to malic acid, pH, and total sugar content. Moreover, *Gluconobacter*, *Lactococcus*, and *Pseudomonas* were closely associated with the production of lactic acid, total acid, and ethanol, respectively. Multiple *Lactococcus* species were critical for synthesizing lactic acid, ethanol, and acetic acid [58].

### 3.4. Analysis Between Key Microorganisms and Volatile Compounds

#### 3.4.1. Identification of Key Microorganisms and Volatile Compounds

**Figure 7:**
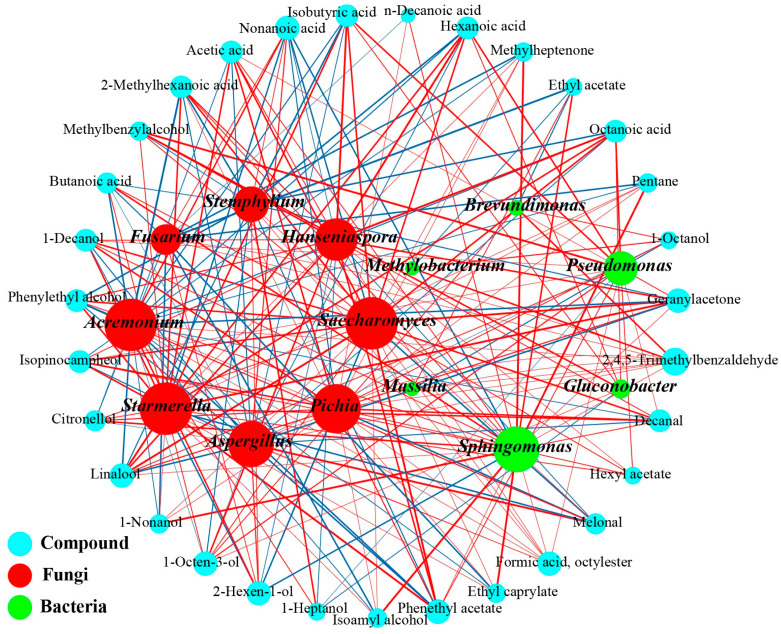
Correlation networks between the microorganisms and aromatic compounds during fermentation. Aromatic compounds and microorganisms are represented by circles of various colors. Positive correlations are shown with red lines, while negative correlations are depicted with blue lines. The strength of the correlation is illustrated by the thickness of the line, with thicker lines representing stronger correlations.

The spontaneous fermentation of wine is a microbial metabolism process, with a close relationship between microorganisms and the generation of aromatic compounds [59]. O2PLS analysis was used to compute variable importance in projection (VIP) values, facilitating the identification of key microorganisms and flavor metabolites throughout the fermentation process. The VIP values exceeded 1.0, indicating the pivotal role of these microorganisms. Specifically, the VIP values for volatile compounds were assessed for each genus of fungi and bacteria; those >1 for both fungal and bacterial groups are highlighted in red and blue, respectively, emphasizing their essential contribution to volatile compound synthesis. In total, 40 genera, comprising the top 20 fungal and 20 bacterial genera identified by average relative abundance, were classified as X variables, while 59 flavor compounds were identified as Y variables. The core functional microbial community and key flavor compounds (VIP > 1.0) consisted of 8 fungal (Figure S7), 10 bacteria (Figure S8), and 34 flavor compounds (Figure S9). Spearman’s correlation analysis revealed significant correlations between these key microbial communities and flavor substances (|r| > 0.60, *p* < 0.01). As shown in Figure 7, the core microorganisms and volatile compounds identified in the fermentation process included 8 fungal and 6 bacterial genera and 12 alcohols, 6 esters, 8 acids, 3 aldehydes, 2 ketones, and 2 others, respectively.

#### 3.4.2. Correlation Analysis Microbes and Flavor

The correlations between the core microbial communities and key volatile compounds during the spontaneous fermentation of wine were investigated (Figure 7). The principal microbial contributors to volatile compound formation include *Saccharomyces*, *Aspergillus*, *Starmerella*, *Pichia*, and *Pseudomonas*. Certain esters and alcohols show positive correlations with yeasts frequently present in wines, notably *Saccharomyces* and *Pichia*. *Saccharomyces* demonstrated substantial positive correlations with 12 aroma compounds, which include formic acid octyl ester, phenethyl acetate, linalool, and geranyl acetone. *Pichia* was a significant producer of various secondary metabolites linked to ester biosynthesis, thereby contributing to floral and fruity aromas [60]. *Starmerella* tolerated relatively high ethanol concentrations and therefore persisted in the mid-to-late stages of fermentation [54]. *Vishniacozyma* also warranted attention due to its presence in grapes from multiple organic vineyards in Xinjiang [61]. While *Vishniacozyma* was not the predominant microbe in the process of wine fermentation, its contribution to the generation of volatile compounds should not be overlooked. Furthermore, this genus generates antimicrobial substances and enzymes that assist in maintaining its ecological niche, potentially linked to the production of aldehydes and acids throughout the fermentation process. The effects of yeast and mold on volatile aromatic substances exhibited contrasting trends. *Saccharomyces* and *Pichia* enhance the formation of most alcohols and esters, whereas *Aspergillus*, *Acremonium*, and *Fusarium* inhibit the synthesis of many volatile compounds. Notably, *Saccharomyces*, *Pichia*, and other yeasts suppressed the formation of nonanoic acid, melonal, and 2-hexen-1-ol. In contrast, *Aspergillus*, *Acremonium*, and *Fusarium* facilitate the synthesis of nonanoic acid and other substances. Among these molds, *Aspergillus* has demonstrated considerable environmental adaptability and resistance to both acidic and ethanol conditions.

Bacterial communities also influence wine aroma and flavor through the production of volatile metabolites [62]. Among the bacterial genera examined, *Lactococcus*, *Massilia*, *Pseudomonas*, *Gluconobacter*, and *Sphingomonas* were associated with the production of phenethyl acetate, 1-heptanol, hexanoic acid, octyl formate, and ethyl lactate, respectively. The results indicated that *Pseudomonas* and *Sphingomonas* were positively correlated with 12 and 17 volatile components, respectively, positioning them as significant contributors to the overall flavor profile. Furthermore, the significant positive correlation between *Pseudomonas* and multiple alcohols observed in this study suggests that *Pseudomonas* possesses a notable capacity for alcohol production. *Methylobacterium* displayed a significant correlation exclusively with ethyl acetate and 1-octen-3-ol, whereas *Gluconobacter*, a member of the acetic acid bacterial group, was solely associated with acids and alcohols. Both *Gluconobacter* and *Methylobacterium* were prevalent during the early fermentation phases but showed a reduction in numbers as fermentation advanced. These species demonstrate a high sensitivity to ethanol, leading to their typical decline during the alcoholic fermentation process [63]. Notably, although the average relative abundances of *Gluconobacter* and *Methylobacterium* throughout the fermentation process were low, these genera showed a positive correlation with an increased presence of aromatic substances.

Significant effects of fungal and bacterial populations on wine characteristics have been extensively documented. Our analysis of the interactions among these microbial communities further underscores their contribution in shaping the wine flavor. Therefore, understanding the dynamic succession of microorganisms and the corresponding changes in their metabolite profiles may contribute to improving wine quality.

## 4. Conclusions

The physicochemical results indicated that the ethanol and acetic acid contents significantly increased over the fermentation period, whereas total sugar, malic acid, and tartaric acid decreased notably. A total of 59 flavor compounds were discovered, with phenethyl alcohol, isoamyl acetate, ethyl acetate, and ethyl hexanoate emerging as the principal aroma-contributing substances. HTS revealed a significant decrease in fungal community diversity accompanied by a significant increase in bacterial community diversity as fermentation progressed. The dominant microbial taxa, identified as *Saccharomyces*, *Starmerella*, *Pichia*, *Pseudomonas*, *Sphingomonas*, and *Aspergillus*, collectively enriched wine flavor complexity through metabolic regulation. The relationships among the microbial community, physicochemical factors, and flavor compounds were analyzed. Notably, *Saccharomyces* was positively correlated with *Starmerella*, *Pseudomonas*, and *Sphingomonas*, whereas *Massilia* was negatively correlated with *Pseudomonas*, *Gluconobacter*, *Pantoea*, and *Curtobacterium*. Correlation analyses showed that total acids and ethanol were the core factors influencing microbial succession. In particular, *Saccharomyces*, *Starmerella*, *Gluconobacter*, and *Lactococcus* were highly correlated with lactic acid, total acid, and ethanol production. These microorganisms not only contribute to microbial community succession but also influence the overall taste and flavor profile of wine. The findings of this study provide essential insights into the microbial succession and metabolic flavor changes that occur during the spontaneous fermentation of Marselan wine in Xinjiang. Future research can combine multi-omics methods to further explore the relationship between microorganisms and metabolites, provide valuable insights into the production of high-quality wine, and advance wine-making technologies.

## Data Availability

The original contributions presented in the study are included in the article/Supplementary Material, further inquiries can be directed to the corresponding author.

## Supplementary Materials

The following supporting information can be downloaded at: https://www.mdpi.com/article/10.3390/foods14060994/s1, Figure S1: Bioplot of the PCA for volatile compounds present in the samples; Figure S2: Composition and classification of volatile compounds in different samples; Figure S3: Alpha diversity indexes (Chao 1 and Shannon) of fungal (A) and bacterial (B) communities; Figure S4: Venn plot of OTUs for fungi (A) and bacteria (B) in samples; Figure S5: Dynamic changes in fungal (A) and bacterial (B) communities of different samples at the phylum level; Figure S6: Relationships of co-occurrence and exclusion among various fungi (A), bacteria (B), and between bacteria and fungi (C); Figure S7: The variable importance value (VIP) plot of fungi in Marselan wine during fermentation; Figure S8: The variable importance value (VIP) plot of bacteria in Marselan wine during fermentation; Figure S9: The variable importance value (VIP) plot of volatile compounds in Marselan wine during fermentation; Table S1: The compositions and contents of volatile compounds in Marselan wine during spontaneous fermentation; Table S2: The richness and diversity indices of fungal and bacterial communities in all tested samples during spontaneous fermentation.
