# Supplementary material for: A Snapshot of Microbial Succession and Volatile Component Dynamics of Marselan Wine in Xinjiang During Spontaneous Fermentation
Source: Foods. 2025 Mar 14;14(6):994. doi: 10.3390/foods14060994 (PMC11941887; doi:10.3390/foods14060994)
Supplement: Supplementary file 1 [file foods-14-00994-s001.zip › foods-3504349-supplementary.pdf]

Supplementary materials for:

# A Snapshot of Microbial Succession and Volatile Component Dynamics of Marselan Wine in Xinjiang during Spontaneous Fermentation

Qingquan Fu [1,2,3,4], Fangfang Wang [1,2,3,4], Tiantian Tang [1,2,3,4], Zimen Liu [1,2,3,4], Lilin Wang [5], Qinglin Wang [1,2,3,4], Xuewei Shi [1,2,3,4] and Bin Wang [1,2,3,4]*

1 Food College, Shihezi University, Shihezi 832000

2 Key Laboratory of Characteristics Agricultural Product Processing and Quality Control (Co-Construction by Ministry and Province), Ministry of Agriculture and Rural Affairs, School of Food Science and Technology, Shihezi University, Shihezi 832000, China

3 Key Laboratory for Food Nutrition and Safety Control of Xinjiang Production and Construction Corps, School of Food Science and Technology, Shihezi University, Shihezi 832000, China

4 Engineering Research Center of Storage and Processing of Xinjiang Characteristic Fruits and Vegetables, Ministry of Education, School of Food Science and Technology, Shihezi University, Shihezi 832000, China

5 Production & Construction Group Key Laboratory of Special Agricultural Products Further Processing in Southern Xinjiang, College of Food Science and Engineering, Tarim University, Alar 843300, China

* Correspondence:binwang0228@shzu.edu.cn; Tel.: +86-0993-2057399

# Content

Content ........................................................................................................ 2
Supplementary Tables ........................................................................................................ 1
Table S1. The compositions and contents of volatile compounds in Marselan wine during spontaneous fermentation ........................................................................................................ 1
Table S2. The richness and diversity indices of fungal and bacterial communities in all tested samples during spontaneous fermentation. ........................................................................................................ 5
Supplementary Figures ........................................................................................................ 6
Figure S1. Bioplot of the PCA for volatile compounds present in the samples. ........................ 6
Figure S2. Composition and classification of volatile compounds in different samples. .......... 6
Figure S3. Alpha diversity indexes (Chao 1 and Shannon) of fungi (A) and bacterial (B) communities. ........................................................................................................ 7
Figure S4. Venn plot of OTUs for fungi (A) and bacteria (B) in samples. ................................ 8
Figure S5. Dynamic change in fungi (A) and bacterial (B) communities of different samples at the phylum level. ........................................................................................................ 8
Figure S6. Relationships of co-occurrence and exclusion among various fungi (A), bacteria (B) and between bacteria and fungi (C). ........................................................................ 9
Figure S7. The variable importance value (VIP) plot of fungi in Marselan wine during fermentation, bar with red color indicated VIP > 1. ........................................................ 9
Figure S8. The variable importance value (VIP) plot of bacteria in Marselan wine during fermentation, bar with red color indicated VIP > 1. ........................................................ 10
Figure S9. The variable importance value (VIP) plot of volatile compounds in Marselan wine during fermentation, bar with red color indicated VIP > 1. .................................................... 10

# Supplementary Tables

**Table S1. The compositions and contents of volatile compounds in Marselan wine during spontaneous fermentation**

| Volatile compounds | RI[§] | Aroma content(μg/L) | | | | | Odor threshold (μg/L) | OAV values |
|---|---|---|---|---|---|---|---|---|
| | | A | B | C | D | E | | |
| **Acetates** | | | | | | | | |
| Ethyl acetate | 612 | 40.9±5.48$^{c}$ | 347.06±21.6$^{b}$ | 323.99±7.87$^{b}$ | 349.86±3.97$^{b}$ | 407.33±24.83$^{a}$ | 7500 | <0.1 |
| Ethyl butanoate | 1037 | nd | nd | 0.92±0.03$^{c}$ | 2.98±0.06$^{b}$ | 24.41±0.74$^{a}$ | 20 | ＞1 |
| Isoamyl acetate | 876 | nd | 0.44±0.03$^{c}$ | 183.26±3.19$^{a}$ | 14.24±0.46$^{bc}$ | 56.53±44.17$^{b}$ | 30 | ＞1 |
| Lactide | 1070 | 2.01±0.36$^{c}$ | 2.15±0.12$^{c}$ | 2.91±0.1$^{b}$ | 1.93±0.19$^{c}$ | 5.08±0.22$^{a}$ | 1600 | <0.1 |
| Ethyl hexanoate | 1230 | nd | 14.45±0.63$^{b}$ | 14.63±1.02$^{b}$ | 16.04±0.03$^{b}$ | 197.53±3.44$^{b}$ | 5 | ＞1 |
| Hexyl acetate | 1011 | 0.26±0.05$^{c}$ | 1.65±0.22$^{b}$ | 4.06±0.19$^{a}$ | 0.57±0.05$^{c}$ | 1.89±0.28$^{b}$ | 670 | <0.1 |
| Ethyl caprylate | 1196 | 0.81±0.03$^{d}$ | 13.72±0.5$^{d}$ | 331.06±66.89$^{c}$ | 533.27±1.04$^{b}$ | 840±10.1$^{a}$ | 580 | ＞1 |
| Formic acid, octylester | 1557 | 2.58±0.56$^{e}$ | 7.76±0.1$^{d}$ | 20.2±0.56$^{a}$ | 11.94±0.52$^{b}$ | 10.42±0.77$^{c}$ | 25482 | <0.1 |
| Ethyl caprate | 1396 | 0.78±0.06$^{d}$ | 4.7±0.33$^{d}$ | 48.42±2.15$^{c}$ | 76.65±5.53$^{b}$ | 203.55±7.01$^{a}$ | 200 | ＞1 |
| Benzyl acetate | 1735 | 0.68±0.06$^{b}$ | 1.2±0.06$^{a}$ | nd | nd | nd | 300 | |
| Phenethyl acetate | 1258 | nd | 11.21±0.12$^{d}$ | 161.83±5.01$^{a}$ | 73.67±1.79$^{b}$ | 21.27±0.59$^{c}$ | 45.5 | 0.1-1 |
| Ethyl laurate | 1595 | 0.04±0.05$^{d}$ | 0.48±0.05$^{d}$ | 4.26±0.16$^{b}$ | 1.4±0.22$^{c}$ | 19.94±0.5$^{a}$ | 500 | <0.1 |

| | | | | | | | | |
|---|---|---|---|---|---|---|---|---|
| Ethyl lactate | 1171 | 0.41±0.12[b] | nd | 8.01±0.1[a] | nd | nd | 53 | |
| **Alcohols** | | | | | | | | |
| 3-Methyl-1-butanol | 1201 | 26.93±0.62[e] | 393.66±23.14[d] | 1413.1±73.76[c] | 2391.51±155.21[b] | 3262.89±90.12[a] | 5000 | 0.1-1 |
| 1-Heptanol | 1452 | 1.76±0.31[a] | 0.69±0.11[c] | 1.06±0.09[b] | 0.62±0.03[c] | 1.23±0.01[b] | 1000 | <0.1 |
| 1-Octanol | 1362 | 3.57±0.24[b] | 1.14±0.14[d] | 5.54±0.27[a] | 1.27±0.06[d] | 2.37±0.06[c] | 120 | <0.1 |
| 3-Methyl-1-pentanol | 1335 | nd | nd | 4.52±0.16[a] | 4.24±0.1[a] | nd | 1000 | |
| Isoamyl alcohol | 1377 | 17.2±0.13[a] | nd | nd | nd | nd | 400 | |
| Cyclohexanol | 880 | 87.27±69.44[a] | nd | nd | nd | nd | 300 | |
| 2-Hexen-1-ol | 852 | 1.5±0.47[a] | 1.04±0.06[a] | nd | nd | nd | 1000 | |
| 2,3-Dimethyl-3-octanol | | 0.75±0.1[c] | 1.62±0.1[a] | 0.96±0.05[b] | nd | nd | 500 | |
| 1-Octen-3-ol | 1224 | 4.06±0.29[b] | 4.31±0.05[b] | 5.94±0.38[a] | 4.53±0.08[b] | 3.1±0.17[c] | 20 | 0.1-1 |
| 2,3-Dimethylcyclohexanol | 1465 | 0.35±0.04[b] | 1.27±0.17[a] | nd | nd | nd | nd | |
| 2-Ethyl-1-hexanol | 1493 | 4.68±0.76[c] | 6.84±0.25[b] | 6.81±0.14[b] | 21.83±0.6[a] | 7.21±0.21[b] | 150000 | <0.1 |
| Linalool | 1546 | 1.09±0.02[d] | 1.76±0.21[c] | 3.78±0.26[a] | 3.54±0.22[a] | 2.96±0.23[b] | 25 | 0.1-1 |
| Isopinocampheol | 1703 | nd | 1.26±0.02[c] | 6.12±0.05[a] | 1.83±0.16[b] | 1.96±0.13[b] | 200 | <0.1 |
| 1-Nonanol | 1654 | 1.07±0.05[e] | 2.19±0.02[d] | 9±0.1[c] | 10.95±0.84[b] | 11.94±0.77[a] | 58 | 0.1-1 |
| Methionol | 1738 | nd | nd | nd | 4.64±0.23[a] | 3.09±0.15[b] | 8 | 0.1-1 |
| 1-Decanol | 1765 | nd | 1.53±0.03[c] | 10.73±0.3[a] | 2.38±0.06[b] | 2.44±0.11[b] | 400 | <0.1 |

| | | | | | | | | |
|---|---|---|---|---|---|---|---|---|
| Citronellol | 1770 | nd | 7.34±0.24$^{d}$ | 31.26±2.44$^{b}$ | 17.94±0.6$^{c}$ | 34.31±0.71$^{a}$ | 50 | 0.1-1 |
| Myrtenol | 1796 | nd | 1.09±0.03$^{a}$ | 0.72±0.05$^{c}$ | 0.49±0.05$^{d}$ | 0.94±0.07$^{b}$ | 7 | 0.1-1 |
| 2,6-Octadien-1-ol, 3,7-dimethyl | 1805 | 0.32±0.05$^{d}$ | 2.49±0.04$^{b}$ | 0.66±0.03$^{c}$ | 3.34±0.17$^{a}$ | nd | 700 | |
| Benzyl alcohol | 1877 | 1.67±0.18$^{c}$ | 8.13±0.34$^{a}$ | 2.19±0.07$^{b}$ | nd | nd | 600 | |
| Phenylethyl alcohol | 1906 | 13.12±1.97$^{e}$ | 190.11±3.43$^{d}$ | 2012.57±107.94$^{b}$ | 2170.48±75.92$^{a}$ | 1353.08±75.6$^{c}$ | 10000 | 0.1-1 |
| Methylbenzylalcohol | 1923 | nd | 1.12±0.03$^{ab}$ | 1.22±0.18$^{a}$ | 1.02±0.01$^{bc}$ | 0.92±0.06$^{c}$ | 363 | <0.1 |
| **Acids** | | | | | | | | |
| Acetic acid | 1459 | 11.05±0.25$^{e}$ | 32.24±1.9$^{d}$ | 85.22±0.61$^{a}$ | 67.27±1.47$^{c}$ | 71.78±0.76$^{b}$ | 4740 | <0.1 |
| Isobutyric acid | 1572 | nd | 1.72±0.09$^{c}$ | 2.36±0.14$^{a}$ | 2.26±0.12$^{b}$ | 1.15±0.08$^{d}$ | 2000 | <0.1 |
| Butanoic acid | 1583 | 0.27±0.05$^{b}$ | 0.43±0.04$^{b}$ | 0.54±0.06$^{ab}$ | 0.95±0.03$^{a}$ | 0.74±0.51$^{ab}$ | 2200 | <0.1 |
| 2-Methylhexanoic acid | 1623 | nd | 2.79±0.23$^{c}$ | 14.66±0.39$^{a}$ | 14.81±1.03$^{a}$ | 7.72±0.07$^{b}$ | nd | |
| Hexanoic acid | 1855 | 6.73±0.42$^{d}$ | 41.56±0.52$^{b}$ | 58.52±1.1$^{a}$ | 56.02±2.53$^{a}$ | 27.36±0.26$^{c}$ | 420 | <0.1 |
| Octanoic acid | 1180 | 5.17±0.25$^{d}$ | 77.54±1.91$^{b}$ | 106.57±2.24$^{a}$ | 77.99±0.88$^{b}$ | 39.21±1.08$^{c}$ | 500 | 0.1-1 |
| Nonanoic acid | 1273 | 1.57±0.22$^{a}$ | 1.12±0.07$^{a}$ | nd | nd | nd | 800 | |
| n-Decanoic acid | 1826 | 0.51±0.03$^{d}$ | 3.56±0.37$^{c}$ | 4.07±0.08$^{b}$ | 3.26±0.09$^{c}$ | 10.56±0.37$^{a}$ | 1000 | <0.1 |
| **Ketones** | | | | | | | | |
| 2,3-Pentanedione | 1065 | 1.63±0.28$^{a}$ | 0.66±0.05$^{b}$ | nd | nd | nd | 3000 | |
| 1-Hepten-3-one | 1224 | 0.61±0.04$^{a}$ | 0.06±0.03$^{c}$ | 0.5±0.02$^{b}$ | 0.46±0.06$^{b}$ | nd | 7 | |

| | | | | | | | | |
|---|---|---|---|---|---|---|---|---|
| Methylheptenone | 1327 | 1.13±0.12$^{d}$ | 1.19±0.07$^{d}$ | 1.54±0.13$^{c}$ | 1.97±0.06$^{b}$ | 2.44±0.16$^{a}$ | 100 | <0.1 |
| Isophorone | 1578 | 3.67±0.08$^{a}$ | 0.9±0.03$^{b}$ | nd | nd | nd | 2546.21 | |
| Geranylacetone | 1453 | 2.21±0.1$^{e}$ | 3.95±0.11$^{d}$ | 15.49±0.72$^{a}$ | 12.98±0.87$^{b}$ | 10.93±0.25$^{c}$ | 60 | 0.1-1 |
| **Aldehydes** | | | | | | | | |
| Hexanal | 800 | 3.47±0.28$^{b}$ | 4.95±0.26$^{a}$ | nd | nd | nd | 10 | |
| 2-Hexenal | 851 | 82.17±1.89$^{a}$ | 36.54±3.95$^{b}$ | 10.85±0.34$^{c}$ | 9.92±0.21$^{c}$ | nd | 30 | |
| Melonal | 1366 | 4.34±0.42$^{b}$ | 4.88±0.5$^{a}$ | 0.66±0.04$^{c}$ | 1.07±0.03$^{c}$ | 1.09±0.03$^{c}$ | 16 | <0.1 |
| Decanal | 1499 | 2.64±0.31$^{d}$ | 6.87±0.16$^{c}$ | 36.12±1.88$^{a}$ | 8.24±0.17$^{c}$ | 17.17±0.23$^{b}$ | 15 | ＞1 |
| Undecanal | 1711 | 0.22±0.18$^{c}$ | 0.44±0.03$^{c}$ | 3.95±0.16$^{a}$ | 0.24±0.01$^{c}$ | 1.46±0.06$^{b}$ | 5000 | <0.1 |
| Pentadecanal | 2041 | 0.13±0.05$^{c}$ | 0.28±0.05$^{b}$ | 2.19±0.1$^{a}$ | 0.23±0.02$^{b}$ | 0.32±0.03$^{b}$ | 430 | <0.1 |
| 2,4,5-trimethylbenzaldehyde | 1896 | 5.16±0.52$^{a}$ | 6.04±0.1$^{a}$ | 3.95±0.27$^{b}$ | nd | nd | nd | |
| **Others** | | | | | | | | |
| Pentane | 1703 | 10.82±0.14$^{e}$ | 36.38±1.14$^{d}$ | 55.68±2.2$^{c}$ | 79.51±3.42$^{b}$ | 162.29±6.98$^{a}$ | 340 | 0.1-1 |
| Olivetol | 1763 | 0.62±0.08$^{a}$ | 0.07±0.01$^{b}$ | nd | nd | nd | nd | |
| 3,5-Di-tert-butylcatechol | 1682 | nd | 1.2±0.16$^{b}$ | 2.83±0.15$^{a}$ | nd | nd | 200 | |
| 2,4-Di-tert-butylphenol | 2309 | 50.78±1.44$^{b}$ | 46.72±2.09$^{c}$ | 107.97±1.47$^{a}$ | 50.03±0.82$^{bc}$ | 48.49±1.55$^{bc}$ | 200 | 0.1-1 |

Italian: A, B, C, D, and E represent samples collected on fermentation days 0, 3, 6, 9, and 12, respectively. Data are expressed as the means ± standard (n = 3). The different lowercase letters in each row indicate a significant difference between the samples ($P < 0.05$). Nd, not detected. RI, retention index; OAV, odor activity value.

**Table S2. The richness and diversity indices of fungal and bacterial communities in all tested samples during spontaneous fermentation.**

| Source | ID | Observed species | Chao1 | Shannon | Simpson | Coverage |
|---|---|---|---|---|---|---|
| | | | **Fungi** | | | |
| | A | 121.33±3.79[a] | 124.48±1.01[a] | 3.59±0.11[a] | 0.87±0.01[a] | 99.99% |
| | B | 91.67±7.51[b] | 92.09±7.01[b] | 2.81±0.25[b] | 0.73±0.05[b] | 100% |
| | C | 69.43±7.09[c] | 62.38±7.17[c] | 1.87±0.09[c] | 0.53±0.01[d] | 100% |
| | D | 62.67±7.37[c] | 62.81±7.61[c] | 2.02±0.19[c] | 0.57±0.04[cd] | 100% |
| Fermentation samples | E | 68±7.55[c] | 68.81±7.61[c] | 2.11±0.2[c] | 0.6±0.04[c] | 99.99% |
| | | | **Bacterial** | | | |
| | A | 98.67±4.04[a] | 99.83±4.97[a] | 6.21±0.07[a] | 0.98±0[a] | 99.06% |
| | B | 100.67±5.69[a] | 115.43±11.29[a] | 5.59±0.09[b] | 0.96±0.01[b] | 95.57% |
| | C | 129.67±38.19[a] | 163.43±73.93[a] | 6.24±0.43[a] | 0.98±0.01[a] | 93.58% |
| | D | 100.33±11.59[a] | 112.59±12.64[a] | 5.8±0.3[ab] | 0.97±0.01[b] | 96.57% |
| | E | 113.67±23.59[a] | 123.01±29.77[a] | 6.16±0.13[a] | 0.98±0[a] | 96.29% |

Each data was expressed as the means ± standard (n=3).

The different lowercase letters in each column indicate a significant difference between the samples ($p < 0.05$).

## Supplementary Figures

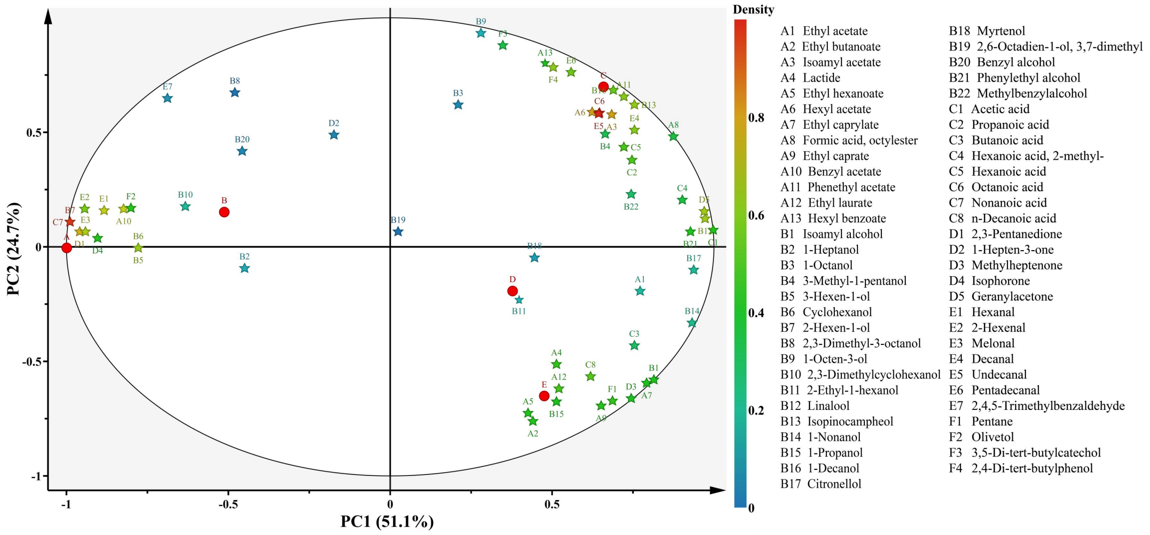


**Figure S1. Bioplot of the PCA for volatile compounds present in the samples.**

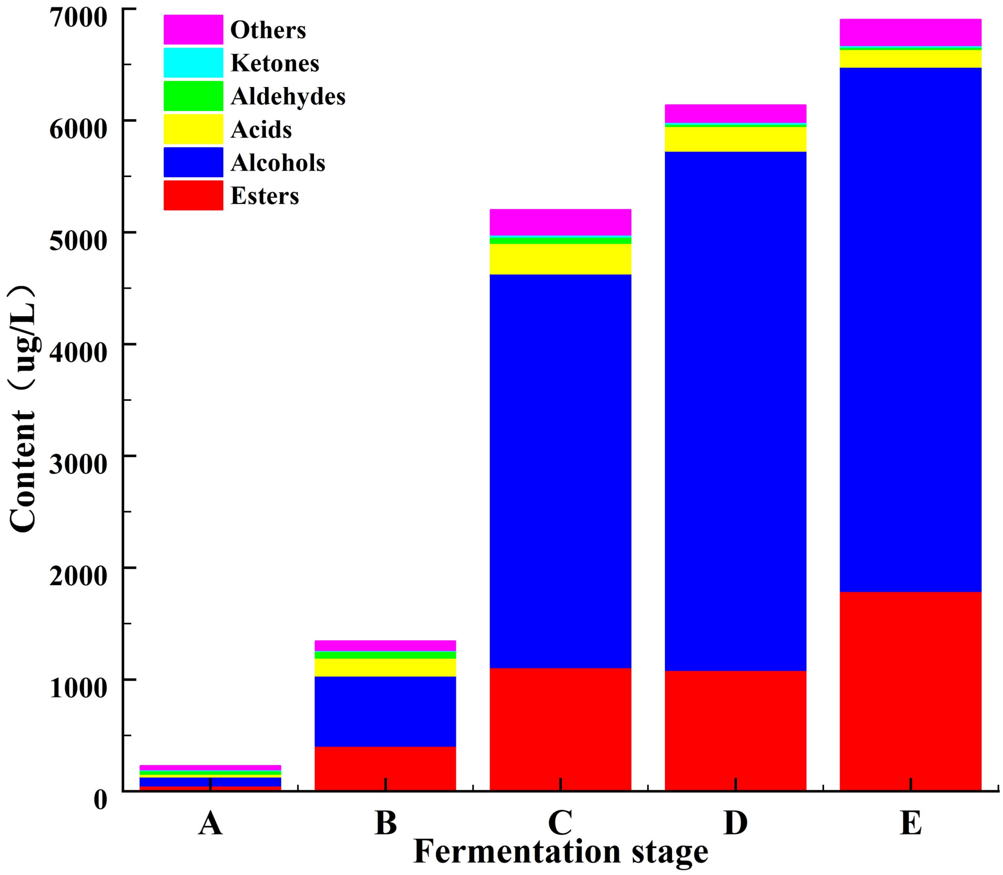


**Figure S2. Composition and classification of volatile compounds in different samples.**

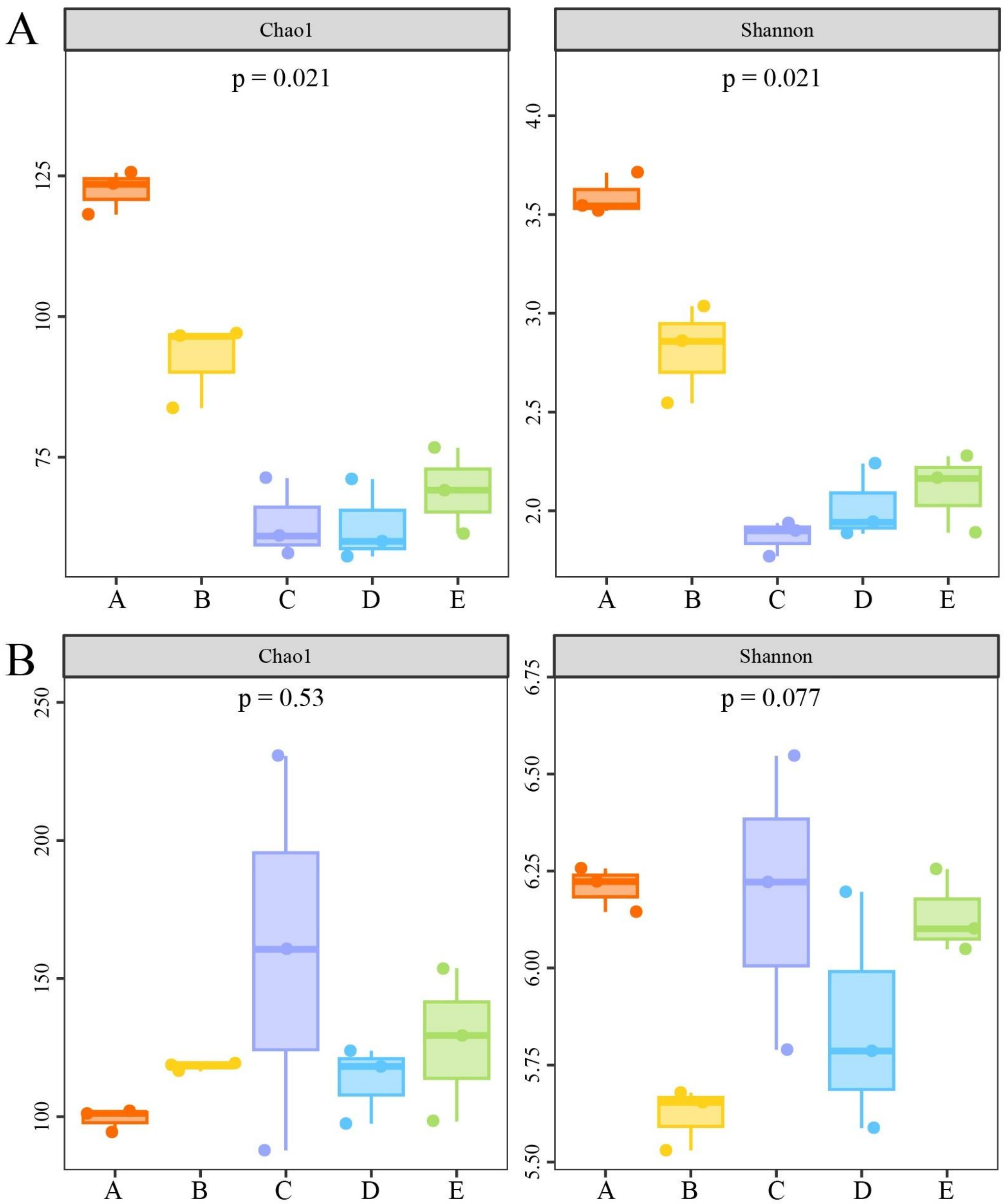


**Figure S3. Alpha diversity indexes (Chao 1 and Shannon) of fungi (A) and bacterial (B) communities.**

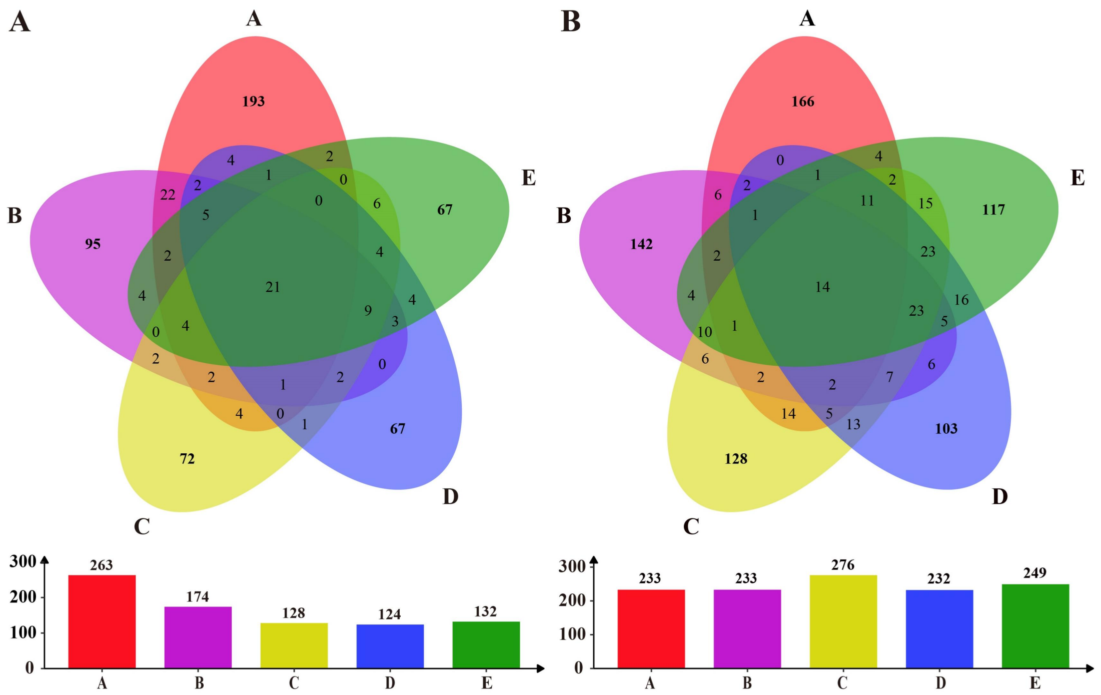


Figure S4. Venn plot of OTUs for fungi (A) and bacteria (B) in samples.

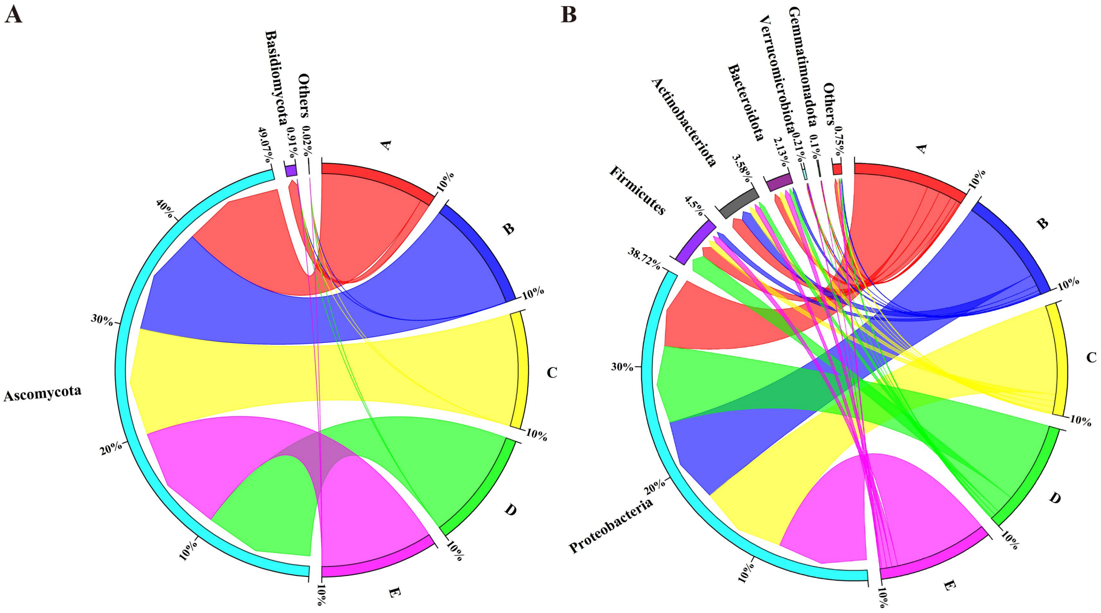


Figure S5. Dynamic change in fungi (A) and bacterial (B) communities of different samples at the phylum level.

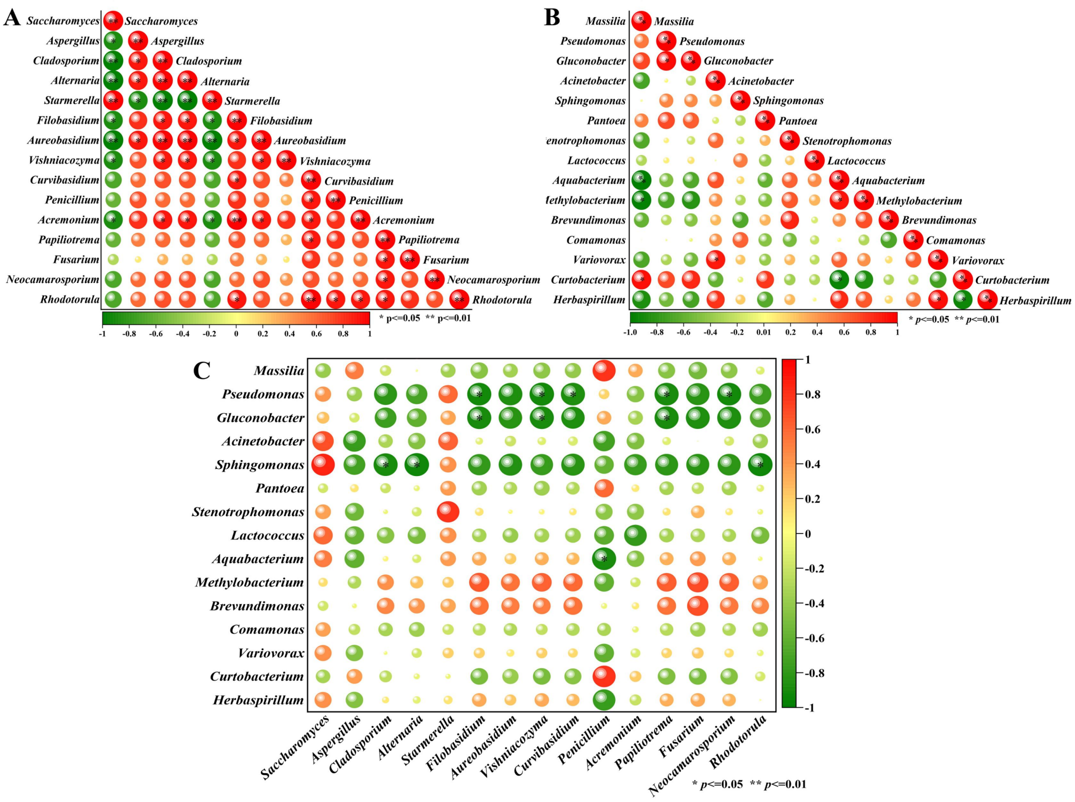


**Figure S6. Relationships of co-occurrence and exclusion among various fungi (A), bacteria (B) and between bacteria and fungi (C).**

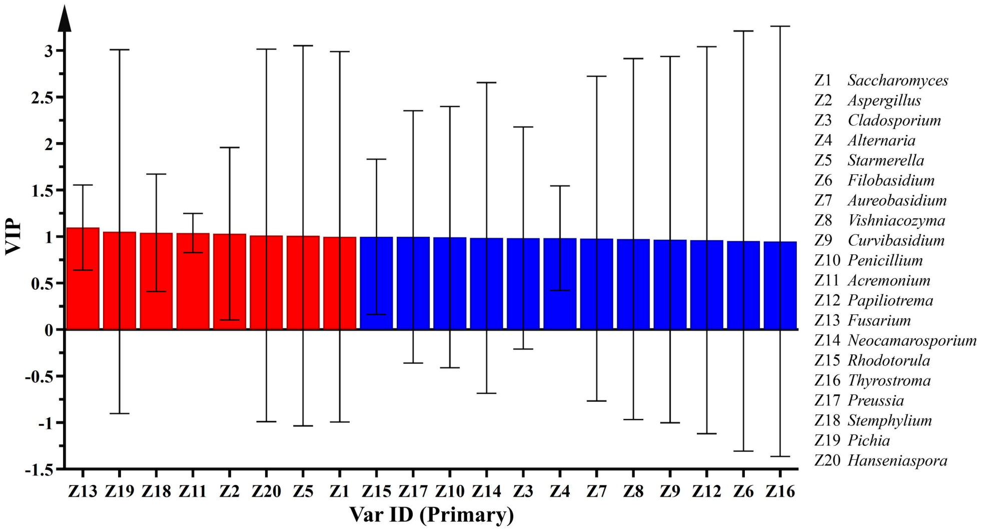


**Figure S7. The variable importance value (VIP) plot of fungi in Marselan wine during fermentation, bar with red color indicated VIP > 1.**

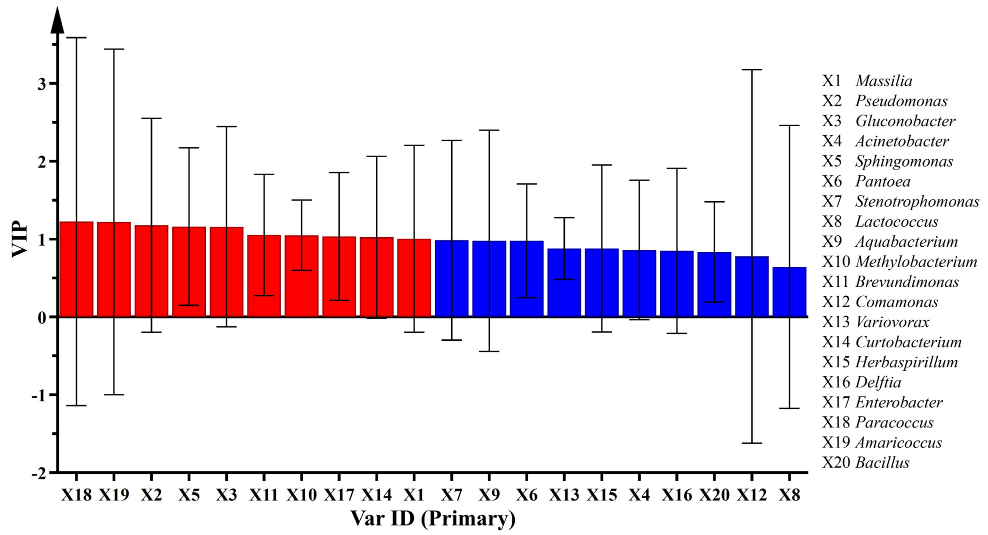


**Figure S8. The variable importance value (VIP) plot of bacteria in Marselan wine during fermentation, bar with red color indicated VIP > 1.**

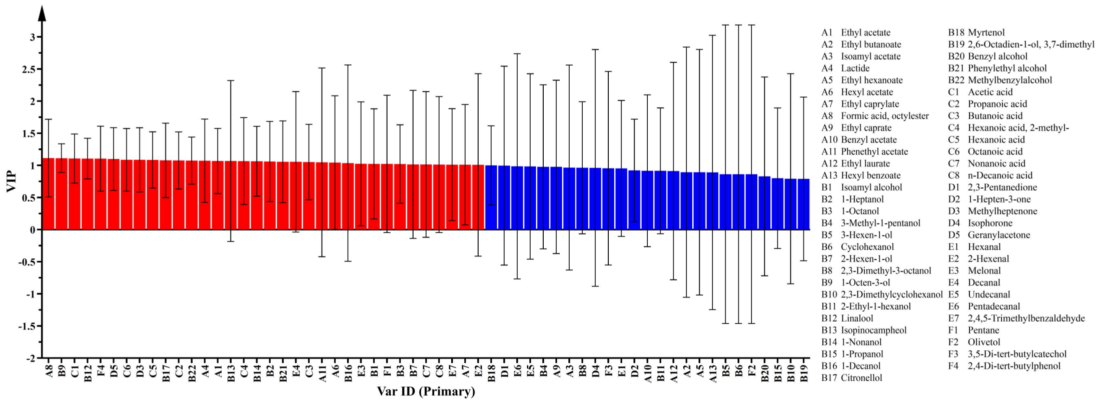


**Figure S9. The variable importance value (VIP) plot of volatile compounds in Marselan wine during fermentation, bar with red color indicated VIP > 1.**
